# Personality in Zoo-Hatched Blanding’s Turtles Affects Behavior and Survival After Reintroduction Into the Wild

**DOI:** 10.3389/fpsyg.2019.02324

**Published:** 2019-10-18

**Authors:** Stephanie Allard, Grace Fuller, Lauri Torgerson-White, Melissa D. Starking, Teresa Yoder-Nowak

**Affiliations:** ^1^Center for Zoo and Aquarium Animal Welfare and Ethics, Detroit Zoological Society, Royal Oak, MI, United States; ^2^Department of Biology, University of Michigan-Flint, Flint, MI, United States

**Keywords:** personality, reintroduction, Blanding’s turtle, conservation, zoo, animal welfare

## Abstract

Reintroduction programs in which captive-bred or reared animals are released into natural habitats are considered a key approach for conservation; however, success rates have generally been low. Accounting for factors that enable individual animals to have a greater chance of survival can not only improve overall conservation outcomes but can also impact the welfare of the individual animals involved. One such factor may be individual personality, and personality research is a growing field. We designed a project to ascertain the presence of personality traits in Blanding’s turtles (*Emydoidea blandingii*), a species of special concern in the state of Michigan, and to assess potential links between traits and post-release success. As hypothesized, the Blanding’s turtles in this study displayed behavioral responses to modified open field tests indicative of distinct personality traits: exploration, boldness, and aggression. Additionally, the personality traits were correlated differently with survival and behavior patterns when the turtles were released into the Shiawassee National Wildlife Refuge. More exploratory turtles had higher survival rates, while neither boldness nor aggression was related to survival. Exploratory turtles were also more likely to travel longer distances after release. The use of muskrat dens was related to increased survival, and both bolder and more exploratory turtles made higher use of this feature. Exploratory and aggressive turtles were found basking outside of water more often, while bold turtles were more likely to be found at the water surface. Both these basking behaviors may increase the risk of predation and may be reflective of a trade-off between the risk and behaviors related to physiological health. Understanding how personality affects behavior and survival post-release can be a critical tool for improving reintroduction success. Zoo animal welfare scientists and practitioners can implement approaches that improve the welfare of individuals within the context of conservation initiatives.

## Introduction

Reintroduction programs in which captive-bred or reared animals are released into natural habitats are considered a key approach for conservation (Bremner-Harrison et al., 2004). Historically, North American zoological parks have played critical roles in the reintroduction of several species extinct or nearly extinct in the wild, including black-footed ferrets (*Mustela nigripes*), California condors (*Gymnogyps californianus*), and the Wyoming toad (*Bufo baxteri*) [Association of Zoos and Aquariums (AZA), n.d.]. Despite these notable examples, a recent literature analysis showed that from 1974 to 2013, zoos and aquariums contributed captive-bred animals to only about 25% of North American reintroduction programs (Brichieri-Colombi et al., 2019). During this time period, zoos contributed the most to amphibian (42%), terrestrial invertebrate (29%), and mammal (19%) programs, with contributions to reptile reintroductions relatively limited at 15% of North American releases (Brichieri-Colombi et al., 2019). With decades of experience in evidence-based breeding and animal management, as well as institutional shifts emphasizing the importance of *in situ* conservation, zoos are well-positioned to increase their contributions to conservation *via* captive breeding and release programs.

Despite their perceived importance as a wildlife conservation strategy, the success rates of reintroduction programs generally have been low (Stamps and Swaisgood, 2007; Swaisgood, 2010; Ewen et al., 2014), and in some cases, large numbers of released captive-bred animals perish (Teixeira et al., 2007). One reason for this is that released individuals may not be prepared to cope with the various challenges they encounter post-release (Beck, 1995; Bremner-Harrison et al., 2004). Thus, animal reintroduction programs naturally include factors that directly impact the welfare of individual animals. However, relatively little discourse has occurred between animal welfare scientists and conservation practitioners (Fraser, 2010). Animal welfare is also rarely monitored or addressed explicitly in published literature about reintroduction programs (Harrington et al., 2013). Incorporating factors that enable individual animals to have a greater chance of survival is not only a welfare goal but can also improve overall conservation outcomes. One such factor may be the impact of individual personality.

The study of personality in animals is a growing field with species studied ranging broadly. In a 2001 review, Gosling identified 187 studies in 64 species, which included mammals, birds, and fish, as well as reptiles, amphibians, arthropods, and mollusks. Perhaps unsurprisingly, animal personality research has focused largely on mammals, ranging from the African striped mouse (*Rhabdomys dilectus*, Joshi and Pillay, 2016) to brown and sloth bears (*Ursus arctos arctos* and *Melursus ursinus inornatus*, respectively, Pastorino et al., 2017), snow leopards (*Uncia uncia*, Gartner and Powell, 2012), African elephants (*Loxodonta africana*, Horback et al., 2013) and a number of non-human primate species including chimpanzees (Freeman et al., 2013), rhesus macaques (*Macaca mulatta*, Capitanio, 1999), and squirrel monkeys (*Saimiri sciureus*, Polgár et al., 2017). Although fewer studies have been devoted to other taxa, some work has been conducted with a variety of reptile species, including snakes, lizards, and turtles. Waters et al. reviewed the existing literature in 2017 and noted that anti-predator behavior in snakes was found to be consistent over time. Additionally, they provided an overview of personality traits found to exist in lizards, including aggression, boldness, exploration, and sociability (Waters et al., 2017).

In turtles and tortoises, as in other species, personality has been explored using a variety of methods. Germano et al. (2017) used the presentation of threatening stimuli to measure boldness and the effect of novel objects on investigative behaviors to measure exploration in desert tortoises (*Gopherus agassizii*). Latency to move from an initial location in an arena was used to assess exploration in red-eared slider turtles (*Trachemys scripta*, Carter et al., 2016) and eastern Hermann’s tortoises (*Eurotestudo boettgeri,* Mafli et al., 2011). Boldness was measured in Spanish terrapins (*Mauremys leprosa*) using the righting response, which is the time it takes an individual to right themselves after being turned over onto their carapace (Ibáñez et al., 2013). A similar method was used to study anti-predator responses in European pond turtles (*Emys orbicularis*, Ibáñez et al., 2018). Kashon and Carlson (2018) measured boldness in eastern box turtles (*Terrapene Carolina*) using the time to emerge from the shell and the time to move after a brief period of confinement. Aggressiveness in eastern Hermann’s tortoises was measured by staging fights between two conspecifics and measuring the amount of time to initiate a fight, rates of biting and ramming, as well as the percentage of fights won or given up (Mafli et al., 2011).

Terminology used in this field of research has been inconsistent (David and Dall, 2016) with terms such as temperament and behavioral style also being used and noted by some to be interchangeable (e.g., Réale et al., 2007). Others have noted that care is needed when using the term personality (e.g., Waters et al., 2017). The use of terms other than personality may be due, in part, to avoiding anthropomorphic implications (Gosling, 2008; Weinstein et al., 2008), resulting in a focus on behavioral patterns without further connections to emotion or cognition in animal personality research. Weinstein et al. (2008) argue that using the term personality more consistently has a number of advantages, including being able to connect work across fields. Definitions of personality also differ, and using a consistent term requires careful attention to the definition being used. For the purposes of this paper, we define personality broadly as behavioral variation between individuals (Carter et al., 2013). Differences in behavior should remain constant across measures, context, and time (Briffa and Weiss, 2012).

In wild animal populations, personality traits have been linked to specific factors impacting individual fitness, such as general health, metabolic rates, parasitism, dispersal, predation, reproductive success, and survival (Smith and Blumstein, 2008). Given these overall relationships between personality and fitness, it is not surprising that personality traits have been linked to post-release survival and behavior in a variety of species in reintroduction programs. Many such studies have focused on traits including exploration, boldness, and aggression. An individual’s ability to disperse, select suitable habitat, and avoid threats in a new environment may be impacted by their personality (Kelleher et al., 2018), and the tendency to explore has been suggested as a critical trait for reintroduced animals (Berger-Tal and Saltz, 2014). Understanding how the behavior of individuals may affect their survivorship has therefore been suggested as an avenue of research (Harrington et al., 2013).

Additionally, individuals with different personality types likely respond differently physiologically, and behaviorally, to stressors (Carere et al., 2010). Differences in coping with stress can have an impact on how animals respond to reintroductions (Merrick and Koprowski, 2017). Given the importance of maximizing the success of reintroduction programs to advance conservation efforts, consideration should be given to how personality traits impact the survivorship of individual animals.

Although methods used to study personality also vary, two main categories are identified: behavior coding and trait rating. Rating of traits by knowledgeable observers has proven to be reliable and practical (Vazire et al., 2007); however, many studies still rely on direct coding of behaviors to assess personality traits (Gosling, 2001; Vazire et al., 2007). One established paradigm for assessing personality in nonhuman animals is the open field test, which involves measuring the behavior of an animal after entry into an open, novel arena (Perals et al., 2017). The parallels between the open field test and the eventual process of releasing captive-bred individuals into new environments suggest that this approach could be especially informative about how individuals with different behavioral traits might fare after release into wild habitats. As a measure of personality, open field tests are typically thought to capture traits related to exploration (Perals et al., 2017) and/or general activity levels (Carter et al., 2013). Additionally, modified open field tests may be used to assess traits such as aggression and sociality, by using mirrors as a proxy for other individuals (see Réale et al., 2007 for review). Behavior under pressure from predators, including simulated predation threats, has been used to measure the degree of boldness in modified open field tests (see Réale et al., 2007 for review).

Understanding the way in which different individuals may respond to environmental factors, both physical and social, may help conservationists tailor release conditions to individual needs in ways that may maximize their success, while improving the welfare of animals involved in releases. As organizations that emphasize both the welfare of individual animals and the conservation of wild populations, zoos are uniquely qualified to bridge this gap between welfare and conservation practice. One potential way to do so is to use the skills of zoo animal welfare scientists and behaviorists to develop behavioral profiles that may predict how particular individuals will respond to conditions they encounter upon release.

### Blanding’s Turtles

Blanding’s turtles (*Emydoidea blandingii*) are a species of special concern in the state of Michigan. The species is considered vulnerable due to degradation and destruction of natural habitat, and populations are declining due to a number of challenges, including road-related mortality. Additionally, lengthy maturation time, as adults do not reach sexual maturity until 14–20 years of age, and nest and hatchling predation also contribute to lower population growth. In 2011, the Detroit Zoological Society began a collaboration with the U.S. Fish and Wildlife Service and the University of Michigan-Flint to headstart this species and reintroduce juvenile turtles into the Shiawassee National Wildlife Refuge, an area that is part of their natural range in Michigan, USA. Headstarting involves the captive rearing of animals until such a time as they are considered less prone to environmental dangers. Headstarting is used in many taxa, although less so for freshwater turtles (Spencer et al., 2017). The Blanding’s turtles in this conservation program were hatched at the Detroit Zoo and released after reaching a carapace length of at least 10.16 cm or 18 months of age. Prior to release, a number of the turtles were outfitted with radio transmitters to monitor behavior and survival rates, work conducted by University of Michigan-Flint researchers. This monitoring took place between June 2014 and November 2015, encompassing the turtles’ first winter hibernation.

### Project Aim

Due to previous correlations between personality types and reintroduction outcomes, and the relatively lesser amount of personality research conducted on reptiles, we designed a project to ascertain the presence of personality traits in Blanding’s turtles and assess potential links between traits and post-release success. We hypothesized that the turtles would display different personality traits based on performance during a suite of modified open field tests. Furthermore, we hypothesized that turtles rating differently on personality traits would have differential survival, in addition to expressing different behavioral patterns and microhabitat choices, post-release.

## Materials and Methods

### Subjects and Housing

The subjects for this investigation were 23 Blanding’s turtles (*Emydoidea blandingii*) from two clutches that were hatched at the Detroit Zoo in Royal Oak, Michigan, USA, as a part of the headstarting program in cooperation with the U.S. Fish and Wildlife Service, Michigan Department of Natural Resources, and the University of Michigan at Flint. In 2012, two gravid females were captured in the Shiawassee National Wildlife Refuge (SNWR) in Saginaw, Michigan (where the headstarted turtles were later released) and brought to the Detroit Zoo to lay eggs. At the zoo, the females were first radiographed to determine the number of eggs and then induced to lay using oxytocin administered at a dose of 10 U/kg intramuscularly.

**Figure 1:**
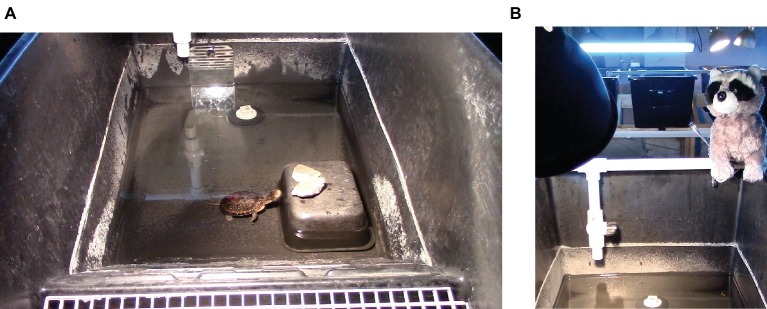
**(A,B)** Enclosure used for housing turtles and as the arena for the four modified open field tests used to assess Blanding’s turtle personality. **(A)** The arena during the second test (mirror test). **(B)** The arena during the fourth test (predator test), with the mock-predator present and other turtle enclosures visible in the background.

Turtle eggs were split into two groups to achieve a ratio of 3:10 males (7:10 females) and incubated for 60–75 days at 28.3°C for males and 30.8°C for females. Hatchlings were raised in groups of 5–6 individuals in TurtleTub^®^ enclosures (Zoo Med Laboratories Inc., San Luis Obispo, CA, USA) measuring 99.1 (l) × 53.3 (w) × 40.6 (d) cm. The enclosures (Figure 1A) were roughly divided into half land area (including a dry ramp from the water to the land) and half water, which was filled to a depth of 7.6 cm for the turtles’ first year of life and 17.8–22.9 cm after that. Water temperature was maintained at 25.5°C, and air temperature ranged from 23.9 to 26.7°C. The water contained plants for the turtles to climb on and a drain plug, and the tanks were connected through a shared filtration system. UV lights were hung 60 cm above the enclosure. The turtles were maintained on a diet of aquatic turtle pellets, blackworms, and krill. They were fed three times a week, and on these days, they were moved into smaller tubs and left there overnight to consume their food.

### Behavior in the Open Field Tests

Behavioral tests were conducted between May 14 and June 10, 2014 when the turtles were 12 months old. To control for time of day, all tests were conducted from 700 to 1,000 h. Turtles were not fed until after testing was completed, which did not require changing their regular feeding time. All tests were conducted in a single experimental arena, a TurtleTub^®^ identical to their home enclosures (Figure 1A). The arena was cleaned and filled with fresh water between tests with different turtles to reduce the presence of olfactory cues from previous trials. The arena contained a hide made from an overturned plastic dish with one side cut out to serve as an exit, a drain plug, and a waterspout hanging down the side of the tank on one side. The tank was otherwise empty with the exception of items added during the open field tests.

Each turtle was tested in four variations of an open field test to explore the consistency of their responses in different contexts. The first test (Simple Open Field Test) consisted of a standard open field test, in which the turtle could freely explore the tank with no other stimuli present. For the second test (Mirror Test), a mirror was placed next to the waterspout on the side of the tank opposite the hide (Figure 1A). For the third test (Food Test), two small (~1 cm) pieces of mealworms were placed in the tank, one on the side containing the hide and the other on the side with the waterspout. For the fourth test (Predator Test), two pieces of worm were added as in the previous test, and a mock predator (a stuffed toy raccoon, Figure 1B) was placed on the top of the outer wall of the tank next to the waterspout.

The following protocol was used for all trials. Each trial lasted 20–23 min and consisted of the four open field tests, each lasting 5 min. The four tests were administered consecutively, in numerical order, to the turtle once it was placed in the arena to minimize turtle handling. Breaks between tests were minimized and consisted only of enough time to add the mirror, raccoon, or worms to the tank. To start a trial, the turtle was removed using gloved hands from its home tank and placed in the arena, and the hide was placed on top of the turtle. The open field test began immediately when the hide covered the turtle. After 5 min, the mirror was placed in the tank and the next test began. After 5 min, the mirror was removed, and the worm pieces were placed in the tank. After five more minutes, two additional worm pieces were added to the tank without removing the pieces from the previous trial, and the raccoon was put in place. The turtle was never removed from the tank or returned to the hide between subsequent tests.

To explore the consistency of the turtles’ behavior across time, each turtle completed a full trial (all four open field tests) on 3 separate days, for a total of 12 modified open field tests per turtle. Trials were separated by 1 week. The order the turtles were tested was randomized at the start of the first week and kept consistent in subsequent weeks, so each turtle was always tested on the same day of the week. Each trial was videotaped for later analysis of behavior.

The behavior of the turtles during the trials was recorded from videos using The Observer XT 12 (Noldus, Wageningen, the Netherlands) on a Microsoft Surface Tablet (Redmond, WA, USA). Three observers coded all the videos, and inter-observer reliability was confirmed as >90% based on percent agreement coding a test video. For each test, the behavior of the turtle and its use of the hide (outside the hide, partially in the hide or in the hide) were simultaneously recorded as separate channels using continuous sampling and the ethogram in Table 1.

**Table 1:** Ethogram for behavioral data collection in modified open field tests.

Behavior	Behavior type	Operational definition
Strike mirror	Event	Turtle hits mirror with head or nose and then immediately moves or is pushed back away from the mirror
Surface	Event	Turtle moves body so that any part of the head is above the water
Eat worm	State	Capture and consumption (including chewing, swallowing, or holding in the mouth) of a worm
Spit worm out	State	Removes worm from mouth
Retract head	State	Retracts at least head and possibly legs as well
Investigate	State	Nosing (physically touching an object with the nose) or stretching (lengthening the neck so that the nose moves within 1 cm of an object); does not include nosing/stretching at enclosure wall
Climb	State	Movement across an object (plug or hide) or vertical movement on a substrate (climbing the wall); for climbing the wall, at least 2 feet are contacting the wall and the turtle is moving
Move	State	Swimming or walking; turtle may briefly pause movement, surface, or nose/contact the wall during this state
Inactive	State	Turtle is not moving around the enclosure; may be moving head to look around an open area, stretching the neck when not in proximity to objects, or nosing the wall during this state
Other	State	A behavior that does not fit into any of the described categories
Not visible	State	Cannot see body or behavior

### Post-release Tracking and Environmental Assessment

SNWR is a 9,800-acre reserve composed of forested wetland and emergent marsh habitats. Headstarted turtles were released in the SNWR at four different sites in June 2014 when they were 22 months old. The first site consisted of open water habitat; the second was dominated by cattails (*Typha* spp.) and duckweed; the third was dominated by willow (*Salix* spp.) and duckweed; and the fourth was characterized by dense cattail habitat. Detailed methods for field monitoring of released turtles can be found in Starking-Szymanski et al. (2018).

Turtle movements were monitored by radio tracking of transmitters affixed to their carapaces. Between the 2014 and 2015 field seasons, each turtle was located between 23 and 44 times (mean ± standard deviation = 37.3 ± 6.8). Home range sizes were estimated using the minimum convex polygon without including release points. Overall movement patterns were described by summing the distances between each point where turtles were located during subsequent tracking events.

When turtles were located, their behavior was recorded as basking, at the water surface, underwater, swimming, on land, or other. Microhabitat factors were also recorded including vegetation type, water depth and temperature, substrate depth, and air temperature. A total of six microhabitat types were identified using these variables by Starking-Szymanski et al. (2018), and these categories were used for further analyses: cattails, lowland forest, muskrat dens, open water, willows, or other floating vegetation.

## Data Analysis

### Exploratory Factor Analysis

For analysis of behavior in the open field tests, the percent of time spent performing all state behaviors and the rates of event behaviors were first calculated for each of the 12 tests. Descriptive statistics were calculated using Microsoft Excel (Redmond, WA, USA).

The analysis used in this study precluded using all the behaviors in the ethogram for personality assignment, so a subset of behavioral variables were selected or calculated (Table 2) that were consistent with operational definitions of personality (or temperament) in nonhuman animals as identified in Réale et al.’s (2007) review. Behaviors linked to exploration and general activity included distance covered in an open field test (Réale et al., 2007), which was operationalized here as the percent of time moving (high exploration) and the percent of time spent in the hide (low exploration) in the simple open field test. Latency to approach novel objects near food sources has also been used as a measure of exploration (Réale et al., 2007), and in this study, this was simplified by measuring the percent of food consumed in the food test. In rodent tests, rearing in an open field test has also been considered an exploratory behavior (Réale et al., 2007). Even freshwater turtles with limited ability to utilize aquatic oxygen can dive for 6–31 min, depending on water temperature (Priest and Franklin, 2002), so we considered the possibility that coming to the surface of the tank could represent an exploratory behavior perhaps analogous to rearing in rodents. Blanding’s turtles are also known to forage while basking at the water surface (Millar et al., 2012), suggesting that surfacing behavior could play a role in exploring the surroundings for food but could also be related to boldness *via* exposure risk.

**Table 2:** Results of the exploratory factor analysis based on the behavior of *n* = 23 Blanding’s turtles in four modified open field tests.

Behavioral variable	Test type	ICC (3, k)	Communalities (extracted)	Exploration (FAC1)	Boldness (FAC2)	Aggression (FAC3)
Percent of time moving	Simple open field	0.54	0.88	**0.91**	0.05	−0.22
Percent of time spent in hide	Simple open field	0.535	0.67	**−0.79**	0.15	−0.14
Rate of surfacing	Simple open field	0.55	0.56	**0.71**	0.16	0.17
Percent of food consumed	Food test	0.09	0.31	**0.51**	−0.20	−0.10
Difference in percent of time moving	Predator-food test	0.24	0.90	−0.14	**0.94**	−0.02
Difference in rate of surfacing	Predator-food test	0.42	0.54	0.07	**0.72**	−0.14
Difference in latency to consume food	Food – predator test	0.28	0.77	−0.08	**0.87**	−0.001
Rate of striking at mirror	Mirror test	0.30	0.84	0.03	−0.04	**0.91**
Latency to strike conspecific (inverse)	Mirror test	0.10	0.96	−0.03	−0.11	**0.975**

*The table shows the rotated factor matrix. Bold scores indicate the component on which the factor loaded*.

To measure boldness, previous studies have examined the latency for an animal to return to a food source after being startled by a predator (Réale et al., 2007). Other studies have presented the predator and food simultaneously; for example, boldness in Hermann’s tortoises (*Eurotestudo boettgeri*) was measured by the experimenter presenting a food object by hand to the tortoise and measuring an index of behaviors including retraction into the shell, latency to approach the hand, and time spent eating (Mafli et al., 2011). In this study, the food and predator were presented simultaneously, but behaviors were indexed based on values from the presentation of food alone to control for activity level and motivation to feed (calculated as predator-food test). Because the turtles would be expected to approach and consume the food more quickly when the predator was not present, we subtracted the latency to consume food in the predator test from the latency to consume food in the food test (Food-Predator Test), so a larger value would theoretically represent a bolder individual.

Finally, agonistic displays and attacks have been used to operationalize aggression in prior studies utilizing mirror tests, and this study employed mirror strikes as well as the latency to approach the mirror, which could be related to either aggression or boldness based on previous studies (Réale et al., 2007). The inverse of the latency to strike the mirror was used so that a higher score would correspond to greater aggression. For turtles that did not strike the mirror at all, maximum latencies were assigned as the inverse of 300 s (the length of the test).

Behaviors from the ethogram (Table 1) not used in further analysis included spitting out the worm, retracting the head, investigating, and climbing. In some cases (e.g., climbing as a measure of exploration and retracting as a startle response related to boldness), these behaviors were considered but were ultimately dismissed on the basis of their repeatability.

Because repeatability is a central feature of personality (Réale et al., 2007), the repeatability of these behaviors across the three testing days within each turtle was examined using intraclass correlation coefficients (ICCs). The ICC analysis was conducted using a two-way mixed model for consistency, and values for the ICC (3, *k*) are reported in Table 2. From the behaviors that had positive ICC values, a subset was then selected that included each of the open field test types while avoiding variables that were highly auto-correlated within each test.

Exploratory factor analysis (EFA) was used to identify turtle personality traits. Historically, many studies of personality have employed principal components analysis (PCA); however, Budaev (2010) argues that EFA is more appropriate for identifying latent, unobservable behavioral constructs such as personality. Although it is commonly believed that very large samples sizes are required for EFA, recommendations for the correct ratio of variables to sample size are not empirically based (Budaev, 2010). In fact, EFA can be appropriate for sample sizes around *n* = 25 when the communalities of the variables are high (Budaev, 2010). Given the current sample size of *n* = 23 turtles, the EFA was conducted with caution, minimizing the number of variables employed. Behavioral variables were chosen that had positive ICC values, relatively high communalities (Table 2) and that represented the range of testing conditions.

To perform the EFA, percentages, rates, or latencies of behavioral variables were averaged for each turtle across the three testing days. Preliminary testing confirmed suitability of this dataset for EFA using the Kaiser-Meyer-Olkin Measure of Sampling Adequacy (0.55) and Bartlett’s Test of Sphericity (*X*^2^_(36)_ = 114.33, *p* < 0.001) (Budaev, 2010). Behaviors were loaded into the EFA using the correlation matrix. Principal axis factoring was used to extract factors, as recommended when the data violate assumptions of normality (Yong and Pearce, 2013). The number of factors was based on the number of eigenvalues >1, and a three-factor solution was confirmed by visual analysis of the scree plot. Final factors are presented using a varimax rotation (Table 2). Given the small sample size, we only accepted factors with relatively high loadings (>0.5). Finally, factor scores were calculated for each turtle using the Bartlett method, which produces unbiased scores that can be compared across factors (Yong and Pearce, 2013).

### Further Analysis of Personality Factors

We used non-parametric Spearman correlations to examine the relatedness of the factor scores from the EFA analysis. Survival was also compared to the proportion of times the turtles were located in specific microhabitats during tracking, behaviors observed at tracking events, and with average body mass across the 2-year study using Spearman correlations.

The remaining outcome variables were analyzed using generalized linear mixed models. For most models, turtle ID was nested within release group. When applicable, the year (2014 or 2015) was used as a repeated statement. The three factor scores were used as fixed effects for all models. Survival status was modeled using binary regression and a logit link function, and in this case, no repeated statement or offset term was used. Turtle mass was measured twice, once before release and again 1 year later. Mass at the 1-year mark was modeled using a normal distribution with an identity link function and turtle nested by release group; there was no repeated statement or offset term. Mass at release was included as a covariate in the model, and interactions between mass at release and each personality variable were also tested in the model. Home range area (MCP estimate) and straight line distance traveled were modeled using normal distributions and identity link functions, and models were offset by the number of tracking events. Counts of behavior and microhabitat locations observed at each tracking event were summed for each year (2014 and 2015) and modeled using negative binomial distributions and log link functions, with counts offset by the ln(number of tracking events). However, the count of tracking points in lowland forest was analyzed using a Poisson distribution and a log link function because a model would not converge with a negative binomial distribution. We were unable to fit a model for use of other floating vegetation; however, this was not a preferred habitat type (Starking-Szymanski et al., 2018).

Degrees of freedom were calculated for all models using a Satterthwaite Approximation. Model fits were compared using Akaike information criterion (AIC), and the repeated statement was modeled using either an unstructured, variance components, or first-order autoregressive covariance matrix, depending on which produced the lower AIC. Models included a random intercept for release group with an unstructured covariance structure, except for the following variables for which a model could not be fit with a random statement: count of at the water surface, count of lowland forest and count of cattails.

Tests of fixed effects and fixed parameter estimates (followed by 95% confidence intervals in parentheses) are reported for results that were significant (*p* < 0.05) or trended towards significance (0.05 ≤ *p* < 0.1). Exponeniated coefficients are presented for binomial and count variables.

## Results

### Results of the Modified Open Field Tests

The EFA analysis produced three factors that cumulatively explained 71.44% of the variance in turtle behavior, with the first factor (FAC1) accounting for 25.12% of the variance, the second factor (FAC2) accounting for 25.08% of the variance and the third factor (FAC3) accounting for 21.24% of the variance. Based on the categories defined by Réale et al. (2007), we identified FAC1 as exploration, FAC2 as boldness, and FAC3 as aggression.

**Figure 2:**
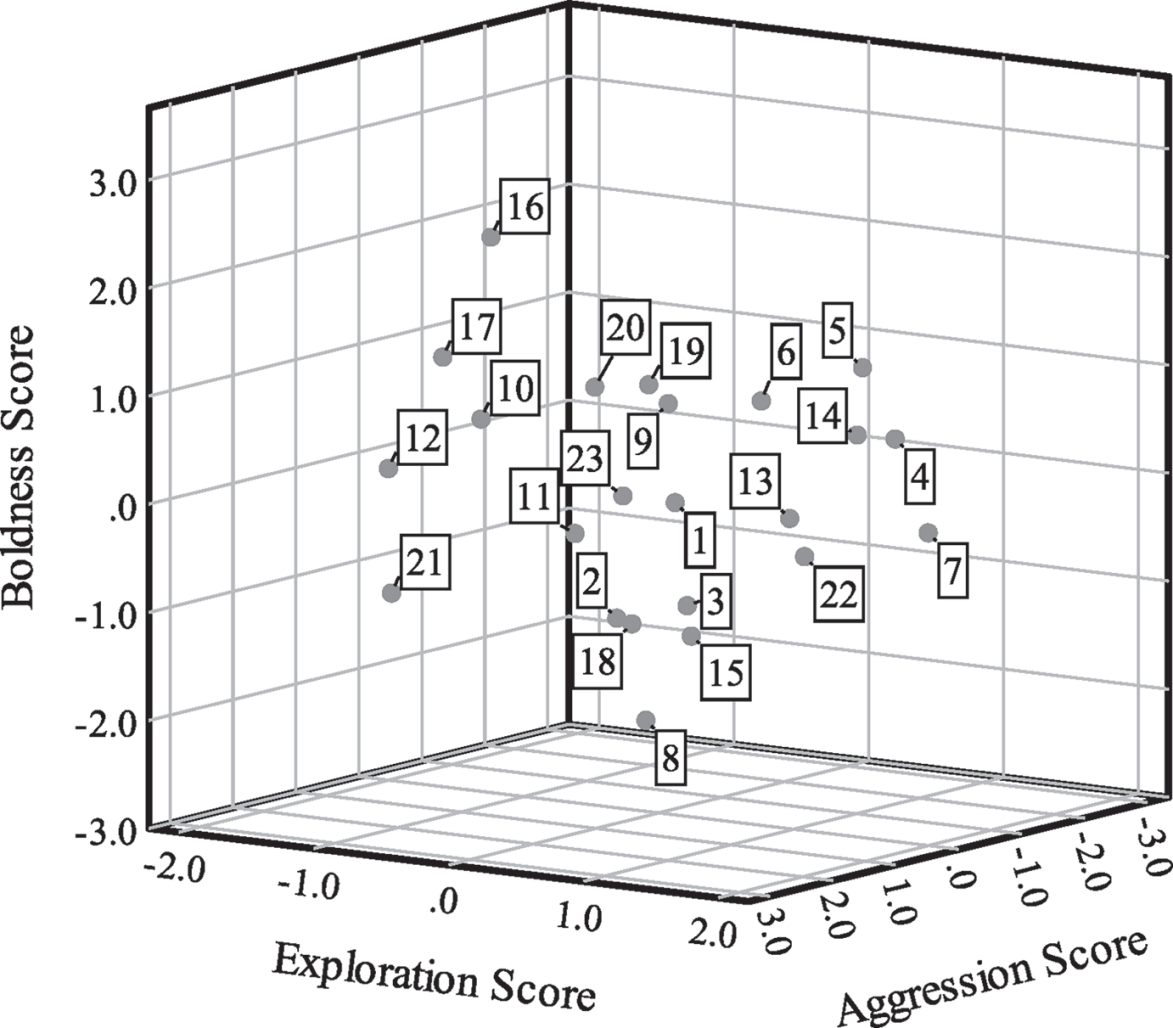
Scatterplot of factor analysis scores showing exploration (factor 1) scores on the x-axis, boldness (factor 2) scores on the y-axis and aggression (factor 3) scores on the z-axis for *n* = 23 Blanding’s turtles. Cases are labeled by turtle number.

Boldness scores were correlated with aggression scores (Spearman’s *ρ* = 0.47, *p* = 0.02, *n* = 23), but bivariate correlations were not statistically significant between exploration and boldness (*ρ* = −0.01, *p* = 0.95, *n* = 23) or exploration and aggression (*ρ* = 0.08, *p* = 0.70, *n* = 23). For exploration, 10 turtles (43.48%) had positive scores and 13 turtles (56.52%) had negative scores. For boldness, 12 turtles (52.17%) had positive scores and 11 turtles (47.83%) had negative scores. Finally, six turtles (26.09%) had positive scores and 17 turtles (73.91%) had negative scores for aggression. Figure 2 presents a scatterplot of the factor scores for the 23 turtles.

### Personality Type, Post-release Behavior, and Microhabitat Selection

**Figure 3:**
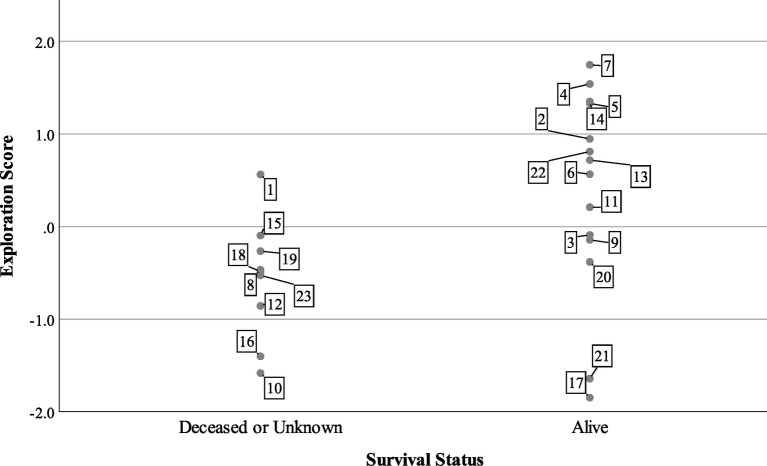
Survival status of *n* = 23 Blanding’s turtles 2 years after reintroduction compared to exploration factor scores. Cases are labeled by turtle number.

At the end of the tracking period in 2015, 14 turtles were alive and being tracked, while one turtle was confirmed dead and eight individuals were missing or of unknown status. Turtles with lower exploration scores were more likely to be dead or missing (Figure 3), but boldness and aggression scores did not predict survival status in the mixed model (Table 3). The model predicted that for every unit increase in exploration score, a turtle was about four times more likely to be alive for 2 years after release.

**Table 3:** Relationships between personality scores and variables related to post-release condition, behavior, and microhabitat selection.

Outcome variable	Exploration (FAC1)	Boldness (FAC2)	Aggression (FAC3)
Survival status (binary)	*F*_1,19_ = 3.67 *p* = 0.07 exp (*b*) = 4.14 (0.88–19.57)	*F*_1,19_ = 0.27 *p* = 0.61	*F*_1,19_ = 0.15 *p* = 0.71
Body mass 1 year after release (g)	*F*_1,14_ = 0.35 *p* = 0.565	*F*_1,14_ = 0.06 *p* = 0.82	*F*_1,15_ = 5.565 **p = 0.03** *b* = −5.39 (−10.27 - -0.51)
MPC home range area (m^2^)	*F*_1,17_ = 0.52 *p* = 0.48	*F*_1,17_ = 0.04 *p* = 0.84	*F*_1,17_ = 0.01 *p* = 0.925
Total straight line distance traveled between tracking points (m)	*F*_1,39_ = 3.01 *p* = 0.09 *b* = 78.145 (−13.00–169.29)	*F*_1,38_ = 0.62 *p* = 0.44	*F*_1,35_ = 0.004 *p* = 0.95
Count of tracking points on land^*^	*F*_1,14_ = 1.13 *p* = 0.31	*F*_1,15_ = 0.29 *p* = 0.60	*F*_1,18_ = 2.49 *p* = 0.13
Count of tracking points basking	*F*_1,16_ = 7.925 **p = 0.01** exp (*b*) = 1.53 (1.11–2.11)	*F*_1,18_ = 2.09 *p* = 0.165	*F*_1,18_ = 5.74 **p = 0.03** exp (*b*) = 1.44 (1.05–1.99)
Count of tracking points at water surface	*F*_1,19_ = 2.71 *p* = 0.12	*F*_1,19_ = 3.78 *p* = 0.07 exp (*b*) = 1.22 (0.985–1.515)	*F*_1,19_ = 1.91 *p* = 0.18
Count of tracking points swimming	*F*_1,18_ = 1.355 *p* = 0.26	*F*_1,19_ = 1.31 *p* = 0.27	*F*_1,18_ = 0.79 *p* = 0.385
Count of tracking points underwater	*F*_1,18_ = 4.955 **p = 0.04** exp (*b*) = 0.955 (0.915–1.00)	*F*_1,19_ = 10.87 **p = 0.004** exp (*b*) = 0.93 (0.88–0.97)	*F*_1,18_ = 0.002 *p* = 0.96
Count of tracking points in cattails	*F*_1,19_ = 5.08 **p = 0.04** exp (*b*) = 0.86 (0.75–0.99)	*F*_1,19_ = 0.86 *p* = 0.365	*F*_1,19_ = 0.40 *p* = 0.54
Count of tracking points in lowland forest^*^	*F*_1,18_ = 1.06 *p* = 0.32	*F*_1,19_ = 3.38 *p* = 0.08 exp (*b*) = 0.50 (0.23–1.10)	*F*_1,19_ = 1.10 *p* = 0.31
Count of tracking points in muskrat dens	*F*_1,18_ = 3.11 *p* = 0.095 exp (*b*) = 1.59 (0.915–2.75)	*F*_1,17_ = 6.37 **p = 0.02** exp (*b*) = 2.21 (1.14–4.30)	*F*_1,18_ = 1.64 *p* = 0.22
Count of tracking points in willow	*F*_1,16_ = 2.06 *p* = 0.17	*F*_1,16_ = 4.695 **p = 0.046** exp (*b*) = 1.41 (1.01–1.96)	*F*_1,16_ = 0.10 *p* = 0.76
Count of tracking points in open water	*F*_1,17_ = 3.96 *p* = 0.06 exp (*b*) = 0.48 (0.22–1.04)	*F*_1,17_ = 5.02 **p = 0.04** exp (*b*) = 2.00 (1.04–3.85)	*F*_1,18_ = 0.05 *p* = 0.82

*Results show tests of fixed effects in generalized liner mixed models. Fixed parameter estimates (followed by 95% confidence intervals in parentheses) are included for results that were significant (in bold, *p* < 0.05) or trended toward significance (0.05 ≤ *p* < 0.1). Exponentiated coefficients are presented for binomial and count variables. Body mass at release significantly predicted body mass 1 year after release and was included as a covariate in the body mass model*.

* *Count of tracking points on land (behavior) and in lowland forest (microhabitat) were analyzed with one outlier excluded (turtle 2), who had the highest factor score for aggression. Turtle 2 also had the highest score for use of lowland forest and the fourth highest score for being observed on land*.

High aggression scores were significantly associated with lower mass 1 year after release (Table 3). However, the most significant predictor of mass at the 1-year mark was mass at release [*F*_1,15_ = 73.53, *p* < 0.001; *b* = 1.18 (0.88–1.47)], and turtles that were heavier at release were heavier 1 year later as well. There was no significant interaction between aggression score and body mass at release (*F*_1,5_ = 1.08, *p* = 0.345) or between release mass and the other personality factor scores. Additionally, there was no relationship between average body mass (for both years) and survival status at the end of the study period (*ρ* = 0.13, *p* = 0.54, *n* = 23).

Turtle movement patterns were impacted weakly by personality variables (Table 3). There was a trend for turtles with higher exploration scores to travel greater distances, as measured by the straight line distance between tracking points (Table 3). Turtles with high (positive) exploration scores had home range sizes about twice those of turtles with low (negative) exploration scores, with a mean MCP estimate of 15,333.42 ± 8,033.71 (standard error) m^2^ for turtles with positive exploration scores and a mean MCP of 7,782.88 ± 2,334.15 m^2^ for turtles with negative exploration scores. However, this difference was not significant in the mixed model analysis (Table 3).

Turtle behaviors during tracking events varied with factor scores (Table 3). Initial analyses showed there was a very strong relationship between aggression score and finding the turtles on land, with more aggressive turtles more often observed on land [*F*_1,19_ = 6.24, *p* = 0.02, exp (*b*) = 11.75 (1.49–92.71)]. However, the high coefficient prompted further inspection of the data, which suggested that one individual (turtle 2), who had the highest individual score for aggression (2.69) and the fourth highest score for the proportion of tracking events on land (0.07), was largely driving this pattern. The relationship between behavior on land and aggression score was no longer statistically significant when this outlier was removed (Table 3). There was no relationship between exploration or boldness score and behavior on land, whether or not turtle 2 was included in the model. Interestingly, turtles more often observed on land were less likely to be alive at the end of the tracking period (*ρ* = −0.50, *p* = 0.02, *n* = 23); however, this pattern does not seem to have been driven by turtle 2, which was alive at the end of the tracking period. Additionally, excluding turtle 2 from models did not significantly change the outcomes for any of the other behavior variables, so turtle 2 was retained in these models. More aggressive turtles were more likely to be found basking (Table 3), but aggression scores did not predict any other behavioral variables.

Turtles with higher exploration scores were less likely to be observed underwater and more likely to be observed basking compared to those with lower exploration scores (Table 3). Turtles with higher boldness scores were much less likely to be found underwater, and there was a trend for more bold turtles to be observed more at the water surface (Table 3). There were no significant relationships between any of the factor scores and the frequency of observing turtles swimming.

Turtle microhabitat usage also showed relationships with personality scores (Table 3). As with behavior on land, initial models showed that turtles with high aggression scores were much more likely to be located in lowland forest [*F*_1,18_ = 17.61, *p* = 0.001, exp (*b*) = 12.0 (3.45–41.67)]. Again, the high coefficient prompted further inspection of the data, which suggested that turtle 2 was having a large impact on this result as well. In this case, turtle 2 not only had the highest aggression score but also the highest proportion of tracking events located in lowland forest (0.75) of all the turtles. Excluding this individual, the model for lowland forest did not show a significant relationship with aggression score (Table 3). However, it is worth noting that despite turtle 2’s relatively moderate exploration score (0.95), when this turtle was included in the model, the relationship between exploration score and lowland forest use attained statistical significance, showing that turtles with higher exploration scores utilized lowland forest less frequently [*F*_1,17_ = 8.17, *p* = 0.01, exp (*b*) = 0.24 (0.08–0.69)]. There were no other microhabitat variables that were significantly related to turtle aggression score, whether or not turtle 2 was retained in the models. Retaining turtle 2 in the models also had minimal effects on fixed effects or parameter estimates related to exploration or boldness scores and usage of other microhabitats; therefore, turtle 2 was utilized in analyses for all the other microhabitat types.

Turtles with higher exploration scores were less likely to be found in cattails and showed a trend to use open water less. However, more exploratory turtles were more likely to be found in muskrat dens (Table 3). Bolder turtles showed a trend to use lowland forest less. However, they were more likely to be found in open water and areas dominated by willow trees and much more likely to be found in muskrat dens compared to peers with lower boldness scores (Table 3).

Microhabitat preferences also showed some relationships with survival (alive or missing/dead) after 2 years. Turtles that were observed in open water a greater proportion of the time were less likely to be alive at the end of the study period (*ρ* = −0.54, *p* = 0.01, *n* = 23). Turtles that spent more time in willow habitat showed a trend towards a decreased likelihood of survival as well (*ρ* = −0.36, *p* = 0.095, *n* = 23). In contrast, there was a positive relationship between the percent of time the turtles were found near muskrat dens and the likelihood of survival (*ρ* = 0.47, *p* = 0.02, *n* = 23).

## Discussion

As hypothesized, the Blanding’s turtles in this study displayed behavioral responses to modified open field tests indicative of distinct personality traits: exploration, boldness, and aggression. Additionally, the personality traits were correlated differently with survival and behavior patterns when the turtles were released into the Shiawassee National Wildlife Refuge.

### Personality Traits

The 23 turtles that underwent behavioral tests were rated on three identified continuums: less to more exploratory, less to more bold, and less to more aggressive. Ten of the turtles showed high exploration, 12 showed high levels of boldness and six showed high aggression. We utilized variations of the open field test to assess these personality traits and selected behaviors for analysis that were reported to reflect these personality traits in previous research (Réale et al., 2007). However, one limitation of our approach was that the number of behaviors we could include in the EFA was constrained by the small sample size. There is a possibility, therefore, that our results could have differed based on the behaviors we selected. For example, we expected that rates of retracting into the shell in the presence of a predator would likely reflect boldness. However, this behavior had a poor ICC value, meaning that individual turtles did not perform it consistently in this context, so we were unable to use it in the factor analysis. The turtles did not obviously direct any behaviors towards the raccoon, so it is possible that they saw the toy raccoon as a novel object rather than a potential predator. If this was the case, the responses in this test could reflect exploration rather than boldness (Réale et al., 2007). However, it is worth noting that the same behaviors (moving percentage, surfacing rate, and percent of food consumed) clustered with exploration when measured in the simple open field and food tests, but the *differences* in these behaviors between the food and predator tests clustered on a different factor—which we identified as boldness. This pattern suggests that the turtles did perceive a meaningful difference between the food and predator tests. The use of multiple measures has been advocated for (Carter et al., 2012), and perhaps future studies could include additional measures to help more definitely identify separate personality traits.

An additional limitation of our experiment is that the four open field tests were always conducted in the same order. For example, turtles that consumed food faster in the predator test (test four) than the food test (test three) may have simply habituated to the experiment, rather than truly showing boldness under threat of a predator attack. We are also unable to account for the habituation and learning processes that would likely occur over the three repetitions of the experiment. We controlled for this by using behaviors with high repeatability in our analysis, but it is possible that order effects and/or habituation could have influenced our results. Despite these limitations, the strong relationships we found between the personality assignments based on the captive tests and the behavior of the turtles after release suggest that the EFA uncovered meaningful individual differences in the turtles’ personalities.

### Personality and Survival

One year post-release, 14 of the turtles were confirmed to be alive. The turtles’ survival was correlated with tendency to explore, with less exploratory turtles more likely to be dead or missing. Similar effects of exploration on survival were found in juvenile desert tortoises (*Gopherus agassizii*, Germano et al., 2017). Neither boldness nor aggression was correlated with survival. Boldness in particular has been found to impact survival positively in other species (e.g., Trinidadian guppies, *Poecilia reticulata*, Smith and Blumstein, 2010; European mink, *Mustela lutreola*, Haage et al., 2017). Contrastingly, boldness was found to decrease survival in reintroduced swift foxes (*Vulpes velox*, Bremner-Harrison et al., 2004), brushtail possums (*Trichosurus vulpecula*, May et al., 2016), and juvenile largemouth bass (*Micropterus salmoides*, Ballew et al., 2017). Carter et al. (2016) found no effect of personality on survival in hatchling red-eared sliders (*Trachemys scripta elegans*). Such findings highlight the need to evaluate the influence of personality at the species level.

More exploratory turtles were also found to have higher body mass, which could be reflective of their ability to locate resources more readily, although in brushtail possums, this was linked to boldness rather than exploration (May et al., 2016). Body mass was not correlated with bold or aggressive traits in this study. Although we found a correlation between exploration and survival as well as body mass, survival and body mass were not correlated. Studies involving other species did find that body mass and survival were linked (Biro and Stamps, 2010; Paterson et al., 2014; Kelleher et al., 2018). This lends support to the impact of personality on survival in these turtles. In other studies, correlations were also found between sex and survival. The sex of the turtles in this study was not determined prior to release, and we therefore cannot make any comparisons based on this factor.

### Personality and Movement

Turtles that rated higher in exploration traveled longer distances post-release. These individuals may have therefore moved to safer or more resource-rich areas, increasing their survival rates. Dingemanse et al. (2003) found that great tits (*Parus major*) did the same. Neither boldness nor aggression were predictors of travel distance. Aggression was associated with dispersal tendency in delicate skinks (*Lampropholis delicata*), but exploration was not (Michelangeli et al., 2017). The underlying mechanism for this tendency could include that more aggressive or bold individuals suppress dispersal in others (Michelangeli et al., 2017). More exploratory wild burbot (*Lota lota*) showed higher rates of movement and larger home ranges (Harrison et al., 2014). Larger home ranges may enable an individual animal to exploit more resources, resulting in more successful individuals.

When hatchling red-eared sliders were monitored after being reintroduced, personality was not found to affect dispersal (Carter et al., 2016). Germano et al. (2017) also found no effect of personality on dispersal in juvenile desert tortoises. However, tendency to travel may also result in non-random distributions of animals with particular personality types. The implications for this include biased population trends that could be more susceptible to environmental changes.

Home range size was not found to differ based on personality type. Although this correlation has been seen in other species (wild burbot, Harrison et al., 2014; brushtail possums, May et al., 2016), these Blanding’s turtles may have benefitted from the types of resources found within their home range but not adjusted the size of their range based on the quality of those resources. Pressure or competition from neighboring individuals may have impacted some turtles’ abilities to expand their home range or move into better habitats.

### Personality and Habitat Use

Based on use versus availability, these turtles displayed personality-dependent habitat selection, which was influenced by the inclusion of particular features. These preferences may have, in turn, impacted turtle survival. None of the turtles were more likely to be found in lowland forest areas. This type of habitat was used less than would be predicted based on availability. In a study of hatchling Blanding’s turtles, Paterson et al. (2012) found that once the turtles moved from terrestrial to aquatic habitats, they tended to remain there. As these turtles were all reproductively immature, using habitat related to travel and access to nesting sites may be less important. Open water was correlated with decreased survival. Interestingly, turtles that scored higher on the boldness scale before release were more likely to be found in open water than other turtles. Fewer resources are available in open water, and this type of space offers little protection from predators. The greater use of open water by bolder turtles in this case could represent a preference antithetical to survival, which is consistent with the finding in other studies that boldness can inhibit survivorship, as in swift foxes (Bremner-Harrison et al., 2004). Open water was also avoided by Blanding’s turtles in a previous study (Millar and Blouin-Demers, 2011) but was actually preferred in another (Ross and Anderson, 1990). Other pressures may affect habitat preferences in different populations.

Cattails were found to be a preferred habitat feature, and as such, all turtles utilized it, showing no differences in personality type. More woody vegetation was also preferred by hatchling Blanding’s turtles in the study by Paterson et al. (2012). For these turtles, bolder individuals were more likely to be found in areas with abundant willow, which was also related to lower rates of survival. Starking-Szymanski et al. (2018) found that overall, the released turtles used this type of habitat less than would be predicted based on availability, suggesting that although willow may provide cover, it may not be a beneficial resource in other ways. Hatchling Blanding’s turtles were more likely to survive when in more structurally complex habitats, such as swamps and marshes, which contain large amounts of vegetation (Paterson et al., 2014). Bogs and wetlands have been found to be preferred by Blanding’s turtles in many cases (for review, see Markle and Chow-Fraser, 2014). It may be that a preference for abundant vegetation overrides selection of more beneficial types of vegetation for some personality types. However, the habitat features/types used to monitor these turtles after reintroduction do not match up perfectly with descriptions used in other studies of Blanding’s turtles and therefore, habitat use comparisons may be affected as a result.

Muskrat dens were used more than expected based on availability (Starking-Szymanski et al., 2018), and use of this feature was correlated with increased survivorship, as seen in juvenile desert tortoises using burrows (Germano et al., 2017). Exploratory and bold turtles were more likely to be found in muskrat dens, and they could have been more willing to enter the dens or more efficient at locating them during their movements. The dens may provide protection from predators, leading to higher survival rates for some of these individuals. These data highlight some of the complexities of linking personality to survival; for example, bolder turtles were more likely to use one type of habitat related to increased survival (muskrat dens) but also preferred another habitat type (open water) related to decreased survival.

### Personality and Post-release Behavior

There were also differences in behavioral tendencies based on the personality type. Bold turtles were more likely to be found at the water surface, suggesting a willingness to surface more readily. This could also be considered a type of basking behavior (McGinnis, 1968; Moll and Legler, 1971). However, aggressive and exploratory turtles were more likely to be found basking out of water, although bolder turtles were not. This is contrary to bold eastern box turtles (*Terrapene ornata*) that maintained higher body temperatures (Kashon and Carlson, 2018) and bold male Namibian agama lizards (*Agama planiceps*) that basked more (Carter et al., 2010). If bold turtles in this case are basking in the water, as indicated by time spent at the water surface, the findings of this study do fit into previous work and highlight the importance of different habitat types for important thermoregulatory behaviors that may be utilized by different personality types. Basking promotes a number of health parameters in ectotherms. Male Spanish terrapins (*Mauremys leprosa*) infected with *Hepatozoon* were more likely to be found basking (Ibáñez et al., 2015). Basking, however, is a more vulnerable position from a predation standpoint. Kashon and Carlson (2018) also found that eastern box turtles displaying higher body temperatures also tended to have more injuries to their shells. There may, therefore, be a trade-off between risk and other factors affecting physiological health. This could be manifested differently between personality types.

As more exploratory turtles were more likely to be found basking, their exposure to predators may also be higher. Convict cichlids (*Amatitlania nigrofasciata*) that spent more time exploring and searching for food were slower to respond to predators (Jones and Godin, 2010). The trade-off between access to resources and potential for predation is an important aspect of personality traits. Although we cannot predict the predator response of more exploratory turtles based on the results of our study, the fact that they are more likely to put themselves in a vulnerable position may also be linked to predation rates. However, less exploratory voles (*Microtus rossiaemeridionalis*) experienced higher predation after being reintroduced (Banks et al., 2002). Consistent impacts should not be assumed when considering how personality affects reintroduced animals.

### Implications for Reintroduction Programs

Understanding how personality affects behavior and survival post-release can be a critical tool for improving reintroduction success. Environmental pressures, including predation, differ between locations, and reintroduced or translocated animals displaying different personality traits may be affected differently. Aggression and boldness are reflective of a proactive coping style (Koolhaas et al., 2007), and these individuals tend to be more successful in stable environments with highly predictable situations (Koolhaas, 2008). Individuals with a more reactive coping style perform better under variable conditions. As individuals differ in their behavioral responses and habitat use, selection of release sites that result in higher likelihoods of survival for a variety of personality types is important. Animals that are less successful in one context may do better in another (Watters and Meehan, 2007). Additionally, individuals display substantial differences in their level of behavioral plasticity (Dingemanse and Wolf, 2013) and thus may not readily adapt to changes in the environment.

One way to safeguard against this type of event would be to release animals representing multiple personality types into all release sites. While this may help at the population level, it will also likely result in negative experiences, including suffering and death, for some of the reintroduced individuals. Acknowledging that not all individuals will fare equally well is also the rationale behind the recommendation to release large numbers of individuals [e.g., Association of Zoos and Aquariums (AZA), 1992; International Union for the Conservation of Nature (IUCN), 2013]. This is a welfare compromise, and the underlying ethical issues still need to be more fully addressed by everyone involved in reintroduction programs. Additionally, especially for species with slower maturation rates or lower reproductive output, each individual has important implications for the success of the species as a whole. If matching individuals with particular personality traits with release sites in which they can be more successful increases survival rates, both the individual animals and the overall populations benefit. Determining personality types present within a conservation breeding population and how those personality traits relate to survivorship should therefore be a consideration within reintroduction programs.

### Zoo Animal Welfare Science and Conservation Initiatives

Increasing overall species survival in reintroduction programs necessitates ensuring that individuals being reintroduced have high survival rates. One could consider this a blurring of individual animal welfare and population or species welfare. Animal welfare science focuses on identifying factors that affect individual animals in captive settings, and many of these factors, such as response to stress and environmental change, also impact animals in the wild. Swaisgood (2010) and Harrington et al. (2013), among others, have advocated for better integration between conservation action and welfare science.

A specific area of animal welfare science that has demonstrated potential as a tool for increasing reintroduction program success is the use and evaluation of enrichment in animals designated for release (Watters and Meehan, 2007; Coelho et al., 2012). Enrichment, when properly designed and implemented, can stimulate a variety of species-appropriate behaviors, such as foraging and investigation. It can also provide varying levels of challenge for animals, which may be lacking in some captive settings (Meehan and Mench, 2007) and may help them cope with the conditions they face post-release. This survival skill-building can improve the success of reintroduction programs (Reading et al., 2013).

Additional tools and methods used by animal welfare scientists should be incorporated into reintroduction programs. Handling and housing animals in ways that minimize stress and exposing captive-bred animals to challenges that may prepare them for wild conditions are potential tools (Fraser, 2010). Overall improvements in captive conditions that promote the development of species-appropriate behaviors and reduce stress and disease can also contribute to effective conservation breeding programs (Greggor et al., 2018). It has also been encouraged to make released animals more comfortable in their release sites, based on the natal habitat preference induction phenomenon (Stamps and Swaisgood, 2007).

Linking *in situ* and *ex situ* conservation initiatives is becoming more common (Minteer and Collins, 2013). Captive breeding programs that release animals back into natural habitats are one example and may be the one practical short-term option to combat declining numbers for some species (Conway, 2011). An increase in the contribution of captive-bred animals by zoos to conservation initiatives has been recommended (Brichieri-Colombi et al., 2019). Collaborations between zoos and other entities involved in conservation programs has also been encouraged, including through the One Plan approach (Barongi et al., 2015). This presents zoo animal welfare scientists with the opportunity to contribute to *in situ* conservation efforts. Utilizing approaches that improve the welfare of individual animals within conservation contexts can better achieve goals of both fields. As suggested by Beausoleil et al. (2019), this could result in a more robust and inclusive field of conservation welfare.

## Data Availability Statement

The datasets generated for this study are available on request to the corresponding author.

## Ethics Statement

The animal study was reviewed and approved by the University Committee for the Use and Care of Animals from the University of Michigan-Flint and the Senior Leadership in Animal Welfare and Management Committee from the Detroit Zoological Society.

## Author Contributions

SA, LT-W, MS, and TY-N contributed to the conception and design of the study. SA, LT-W, and MS collected the data. GF performed the statistical analysis. SA and GF wrote the manuscript. All authors contributed to the manuscript revision, read, and approved the submitted version.

### Conflict of Interest

The authors declare that the research was conducted in the absence of any commercial or financial relationships that could be construed as a potential conflict of interest.
